# Electric-field-induced self-assembly of chlorophyll enables extraction and stabilization

**DOI:** 10.3389/fnut.2026.1844456

**Published:** 2026-07-07

**Authors:** Yangbin Wang, Jingzhi Xue, Shuyu Wang, Yixiao Liu, Fangwei Li, Mingyong Zeng

**Affiliations:** 1 State Key Laboratory of Marine Food Processing and Safety Control, College of Food Science and Engineering, Ocean University of China, Qingdao, China; 2 Sanya Institute of Oceanography, Ocean University of China, Sanya, China

**Keywords:** charge transfer, chlorophyll, electric induction, sustainable extraction, thermal stability

## Abstract

**Introduction:**

Chlorophyll (Chl), a natural photosynthetic pigment, is limited in large-scale applications by costly organic solvent extraction and poor stability under light and heat.

**Methods:**

This study explores an electric induction method to promote Chl aggregation, forming stable aggregates that protect and extract Chl. By controlling induction time, aggregates of varying sizes and quantities are generated.

**Results:**

Quantum chemical calculations on Chl derivatives show that electric induction causes Chl molecules to move toward the positive electrode, forming aggregates that exhibit J-type-like characteristics through intermolecular charge transfer (CT). Chlorophyll self-aggregation markedly enhanced the thermal stability of Chl. The findings indicated that the highest retention rate was observed in the 30 min group, which improved by 78.43% compared to the control group.

**Discussion:**

This efficient extraction method preserves Chl's structure, enhances its thermal stability, and reduces organic solvent use, providing a sustainable approach for Chl extraction and protection.

## 1 Introduction

Chl is widely distributed in plants, algae and microalgae (1). With an estimated annual production of 1.2 billion tons, it is the most abundant photosynthetic pigment on Earth (2). Its anti-inflammatory, antiviral and blood glucose-regulating properties have also garnered significant attention (3–5). Due to their excellent color ability and nutritional benefits, Chl and its derivatives are increasingly utilized in the food industry. However, its large-scale application faces several challenges: the inherent water insolubility of Chl limits its use in aqueous-based products; the extraction process requires large amounts of organic solvents, raising production costs and safety concerns; and poor light/heat stability causes structural changes and color fading.

Microalgae have become an excellent source of natural compounds because of their low land occupation and short growth cycle (6). The main approach for extracting Chl from microalgae is the organic solvent method, primarily using solvents such as acetone, methanol, and petroleum ether. Various auxiliary techniques (e.g., pulsed electric fields, ultrasound, and microwaves) can enhance extraction yields and preserve Chl activity (7), but organic solvent consumption remains high. The resulting large quantities of polluted wastewater further increase treatment costs (8). Non-thermal physical technologies have emerged as promising alternatives for natural pigment extraction (9).

Many approaches have been explored to improve Chl thermal stability (10, 11). The current conventional approach is to use metal ions such as Cu^2+^ to replace the Mg^2+^ on the porphyrin ring of Chl (12), thereby improving Chl color stability during thermal processing. However, the safety of metal Chl complexes remains insufficiently guaranteed (13). Meanwhile, the extensive use of synthetic colorants has raised concerns regarding their potential health risks (14).

Self-assembled aggregates of Chl can greatly improve its stability (15). Sodium chloride or high concentrations have been shown to promote Chl aggregation, offering possibilities for enhancing stability without introducing new substances or altering Chl's intrinsic structure (16). If the increase in aggregate size and quantity leads to macroscopic visible precipitation, it could facilitate its extraction and separation. Therefore, there is a need to investigate conditions that induce Chl aggregation while allowing control over aggregate size. Recent studies have shown that pulsed electric fields can influence chlorophyll aggregates (17, 18), providing insights into electric field-chlorophyll interactions.

Chl efficiently absorbs light quanta, facilitating the reversible release and uptake of electrons, thereby serving essential functions in the conversion of light energy into chemical energy. When orderly assembled, Chl can function as an effective conductive material (19), with the principle lying in intermolecular charge transfer (CT) (20). Based on this principle, this study explores whether electrical induction can trigger intermolecular CT between Chl molecules, potentially enhancing self-aggregation and leading to aggregate formation.

This study aims to: (i) investigate the impact of electric induction on Chl self-aggregation; (ii) elucidate the molecular mechanism using quantum chemical simulations; (iii) evaluate the thermal stability of the formed aggregates; and (iv) assess the potential of this method for Chl extraction and stabilization. This study is expected to provide a novel approach for the extraction and stability enhancement of natural colorants.

## 2 Materials and methods

### 2.1 Preparation of Chl

Chl was extracted from Spirulina (supplied by Hainan Xin Daze Biotechnology Co., Ltd.), which was used as the main material for this study. The extraction approach was modified according to the Wang et al. (21). Briefly, 200 g of Spirulina powder was placed in 200 mL of absolute ethanol and 400 mL of petroleum ether. After thorough mixing, the sample was homogenized using a homogenizer for 5 min. The homogenized sample was then subjected to refrigerated centrifugation at 4 °C and 8,000 g for 10 min. The supernatant was collected after removing the precipitate, subjected to vacuum filtration, and then transferred to a separatory funnel. An equal volume of water was added, shaken, and left to stand for phase separation. The upper organic phase (petroleum ether layer) was retained. The washing step with an equal volume of water was repeated until the lower aqueous phase became colorless. The upper organic phase was collected and concentrated to approximately 200 mL using a rotary evaporator at 36 °C to obtain the crude Chl extract. The crude extract was further purified by column chromatography using neutral alumina as the stationary phase. Carotenes, xanthophylls, and Chl were sequentially eluted with petroleum ether–acetone (v:v = 9:1), petroleum ether–acetone (v:v = 7:3), and n-butanol–ethanol–water (v:v:v = 3:1:1), respectively. The Chl eluate was concentrated using a rotary evaporator at 45 °C, and the purified Chl solution (purity 86.6%) was stored at 4 °C in the dark.

### 2.2 Determination of Chl a content

The column chromatography purification effectively removes carotenes and xanthophylls, and the purity of the final Chl fraction is 86.6%, with Chl a being the absolutely dominant component. Microplate reader (Cytation5M, BioTek, USA) was used to measure the Chl a content. The Chl a content and retention were calculated based on absorbance using the following formula (21).


$$\begin{array}{l} \text{Chl a concentration }\left(\text{mg}/\text{L}\right):\text{ Ch}{\text{l}}_{\text{a}}=\\ \;12.71\times {\text{A}}_{663}-2.59\times {\text{A}}_{645} \end{array}$$ (1)

### 2.3 Exploration of Chl aggregation with electric induction

#### 2.3.1 Experiments of Chl aggregation with electric induction

Chl (15 mg/L) was dissolved in 5 mL ethanol/water (v/v = 1:1) and stored at 4 °C in the dark. Two copper rods (provided by Jiangsu Biling Copper Co., Ltd.), each with a diameter of 0.5 mm and a length of 10 cm, were used as positive and negative electrodes, respectively. The two rods were inserted vertically and parallel into the solution with a spacing of 5 mm between them. A 1.5 V battery (provided by Nanfu Battery Co., Ltd.) was used as the power source and connected to the electrodes, with 1 cm of each electrode submerged in the solution. Changes in the solution were observed throughout the electrification process. Lycopene and β-carotene standards (purity ≥ 96%, purchased from Shanghai Macklin Biochemical Co., Ltd.) were prepared and subjected to electric induction under the same conditions as those used for Chl. Specifically, each standard was dissolved in 5 mL ethanol/water (v/v = 1:1) at a concentration of 15 mg/L, stored at 4 °C in the dark, and subjected to the identical electrode configuration and voltage parameters as described above.

#### 2.3.2 Analysis of spectral

Microplate reader (Cytation5M, BioTek, USA) was used to analysis spectral of Chl solutions. The absorption spectrum was measured with slight modifications to the method described by Kang et al. (22). The absorption spectrum's wavelength range was between 300 and 700 nm. The fluorescence measurement method was based on the approach described by Merzlyak et al. (23) with minor modifications. The excitation wavelength was set to 436 nm (24) and the fluorescence emission spectra were recorded between 500 and 700 nm. All measurements were performed at room temperature using a 96-well plate, with the microplate reader's slit width set to 10 nm.

#### 2.3.3 Observation by inverted fluorescence microscopy

The fluorescence microscope (DMi8, Leica, Germany) was used to view Chl solution. A constant 10 μL of solution was used and the GFP light source was continuously employed (25). Fluorescent images were captured with a computerized imaging system and the entire experiment was carried out in a dark setting.

#### 2.3.4 Experiments of the aggregation of electro induced Chl at the positive electrode

Chl (15 mg/L) was dissolved in 20 mL ethanol/water (v/v = 1:1) and transferred into the apparatus as shown in the Supplementary Figure S1. Two copper rods, each with a diameter of 0.5 mm and a length of 10 cm, were used as the positive and negative electrodes, inserted at each end of the apparatus. The distance between the electrodes was maintained at 1 cm, and the immersion length was controlled at 1 cm. After 2 h of electric induction, 200 μL of the Chl near the bottom right of both the positive and negative electrodes was collected for absorption spectrum analysis. The experiment was repeated three times.

The copper rod as the positive electrode was then rinsed with 10 mL of anhydrous ethanol to collect the substances that detached from it, which were designated as the samples from the positive electrode. A pure Chl ethanol solution (15 mg/L) was prepared as the standard solution (Chl was extracted as described in Section 2.1). Both samples from the positive electrode and pure Chl ethanol solution were analyzed using absorption spectrum. The experiment was repeated three times.

#### 2.3.5 Experiments of electric induction sodium copper chlorophyllin (SCC) and phytol (PYT)

Prepare 5 mL of 15 mg/L SCC (provided by Shanghai Macklin Biochemical Technology Co., Ltd) ethanol/water (v/v=1:1) solution stored at 4 °C in the dark. The electric induction approach was performed as described in 2.3.1. Fluorescence intensity was measured as an indicator of SCC aggregation. The peak of the fluorescence emission spectrum obtained at an excitation wavelength of 402 nm was located at 670 nm, and the peak of the fluorescence emission spectrum obtained at an emission wavelength of 670 nm was located at 402 nm (As results in the Supplementary Figure S2). Thus, the excitation wavelength of SCC was determined to be 402 nm and the fluorescence emission spectra ranged from 550 to 700 nm.

PYT (15 mg/L) (provided by Shanghai Macklin Biochemical Technology Co., Ltd) was dissolved in 5 mL ethanol/water (v/v = 1:1) and stored at 4 °C away from light. The electric induction approach was performed as described in 2.3.1.

#### 2.3.6 Detection of conductivity

Conductivity of the samples was measured using a conductivity meter (DDSJ-308F, INASE Scientific Instrument Co., Ltd., China). 0.01 mol/L KCl was used for calibration. After calibration, the electrode of the meter was immersed in the sample to initiate the measurement, and the conductivity displayed on the screen were recorded.

### 2.4 Experiments of Chl thermal stability after electrical induction

Prepare 25 mL of 15 mg/L Chl ethanol/water (v/v=1:1) solution, take 5 mL and divide into 5 bottles. As in 2.3.1, conduct electric induction for 0 min, 15 min, 30 min, 45 min and 60 min on the 5 groups of solutions. Measure the UV absorption spectra before and after electric induction. Take 1 mL of each group of solutions, place them at 80 °C and heat in the dark for 2 h, measure the UV absorption spectra and Chl content changes before and after heating. Repeat the experiment 3 times.

### 2.5 Quantum chemistry calculations

Gaussian 16 (A.03) was utilized to perform computations for geometric optimization, vibration analysis and excited state calculations of the molecular structure (26). Quantum chemical calculations were performed employing Density Functional Theory (DFT) and Time-Dependent Density Functional Theory (TD-DFT). The geometries were optimized with the B3LYP functional and the 6-31G (d, p) basis set (27) and none of the optimized structures exhibited imaginary frequencies. For the computation of excited states, the CAM-B3LYP-D3 (BJ) functional along with the 6-31G (d) basis set was applied (28). The solvent parameters of eps were 51.5 and epsinf was 1.81037025. Hole-electron analysis was performed using Multiwfn 3.8 (29, 30). The molecular structure model and its isosurface plot were generated using Visual Molecular Dynamics (VMD) software.

### 2.6 Statistical analysis

Experimental data were expressed as “mean ± standard deviation” and the significance of the experimental data was analyzed using analysis of variance of SPSS Statistics 21. The significance level p is 0.05, and p < 0.05 indicates significant difference. Use Origin 2021 software to draw these graphs.

## 3 Results and discussion

### 3.1 Effect of electric induction on solution state, Chl content and spectrum

**Figure 1:**
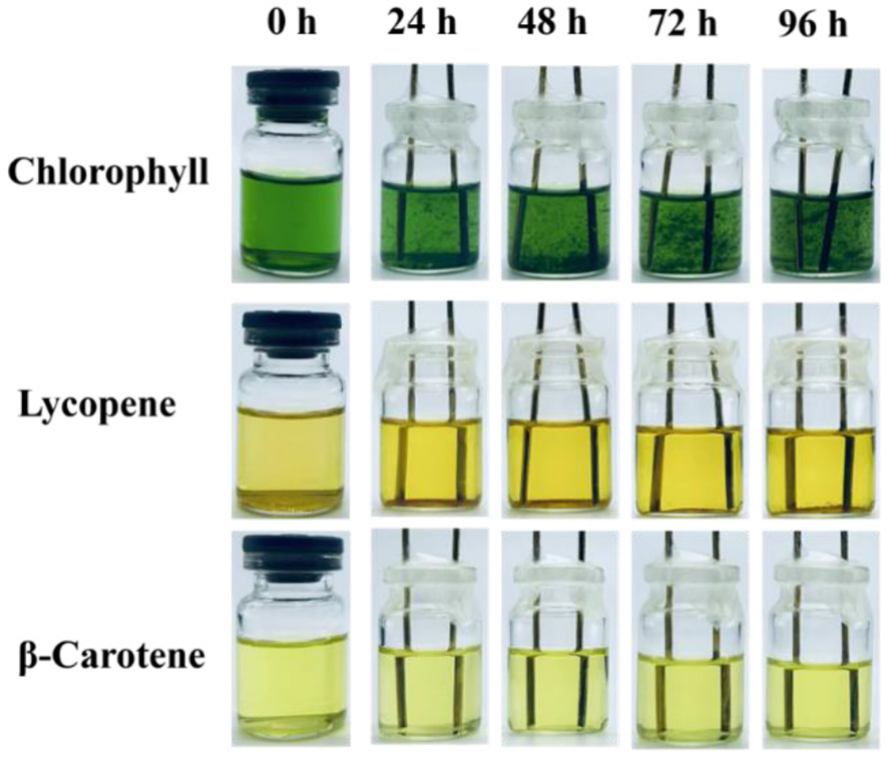
Chl, lycopene and β-carotene solution with electric induction during the 96 h.

**Figure 2:**
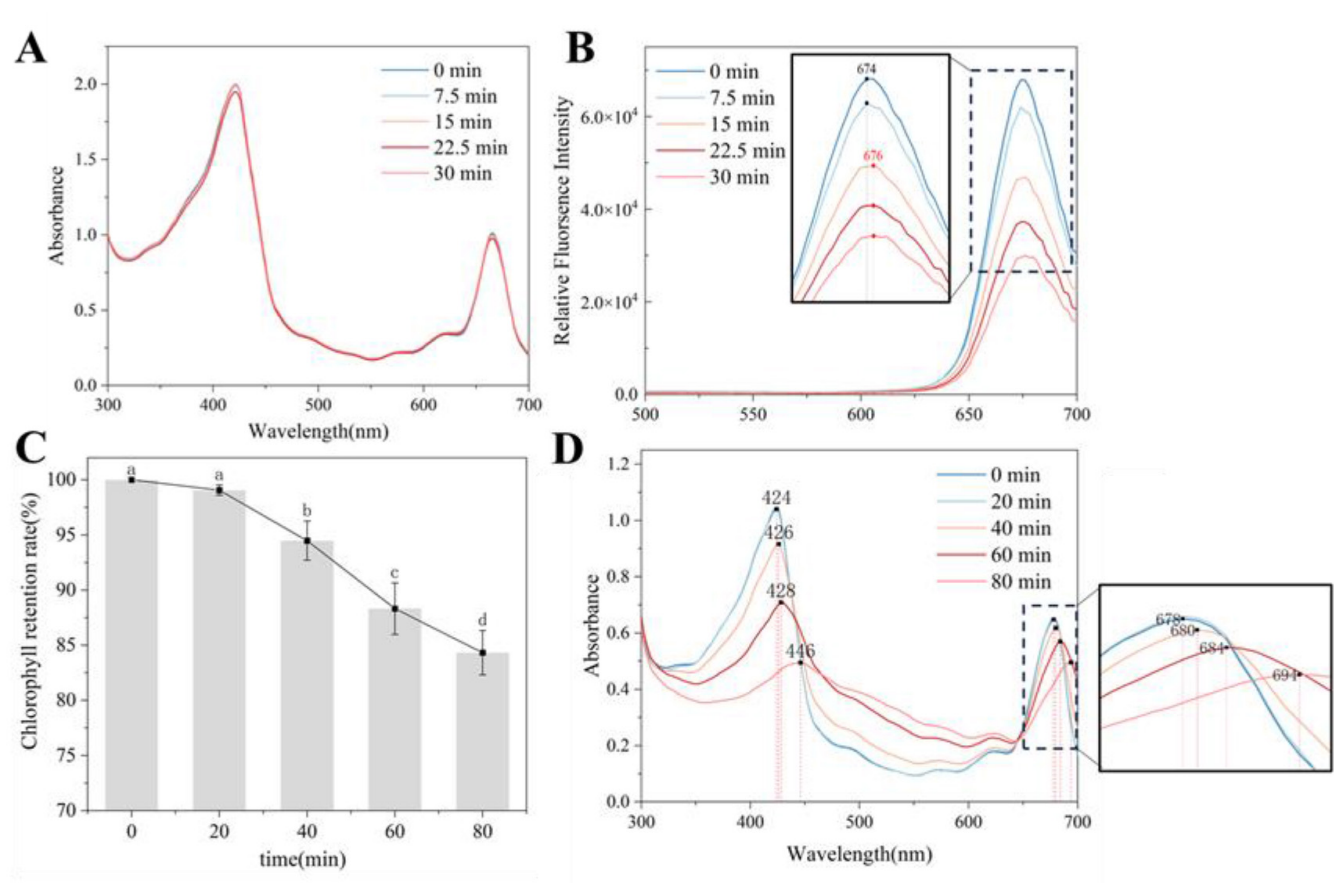
Chl with the electric induction: UV absorption spectra **(A)** and fluorescence emission spectra **(B)** of the Chl in the first 30 min of electric induction; concentration **(C)** and UV absorption spectra **(D)** of Chl in the first 80 min.

The porphyrin ring structure of Chl is a conjugated carbon ring, closely related to the electric conductivity properties of Chl. In addition to Chl, two other pigments which both have linear conjugated structures commonly found in microalgae, lycopene and β-carotene, were also selected as a comparison. With 24 h of electric induction, a visible precipitate formed exclusively in the Chl sample, while no significant changes were observed in the others (Figure 1). Moreover, as the duration of electric induction increased, the amount of precipitate in the Chl solution visibly increased, whereas the other two pigments remained unchanged. The extended 96 h induction experiment was performed at a deliberately low voltage (1.5 V). Our findings indicate that the electric field strength directly determines the rate of Chl aggregation: a stronger electric field leads to faster aggregation. In practical industrial production, a higher voltage can be used to achieve rapid aggregation within a short time, thereby avoiding thermal effects. However, in this study, we deliberately lowered the voltage to 1.5 V to slow down the aggregation process, allowing us to capture and record the detailed phenomena occurring during electric induction. Throughout this extended period, the temperature increase was minimal due to the low voltage and the ethanol/water solvent system. As shown in Figure 2, the key aggregation events occur within the 1 h of induction, confirming that short induction times are sufficient for practical applications. Therefore, thermal effects on Chl aggregation are not a primary concern in this mechanistic study. The experimental observations confirm that prolonged electric induction (up to 96 h) leads to the gradual precipitation of Chl, potentially facilitating its separation from lycopene and β-carotene. This implies that the conjugated carbon ring structure of Chl could be a critical factor in its precipitation under electric induction.

**Figure 3:**
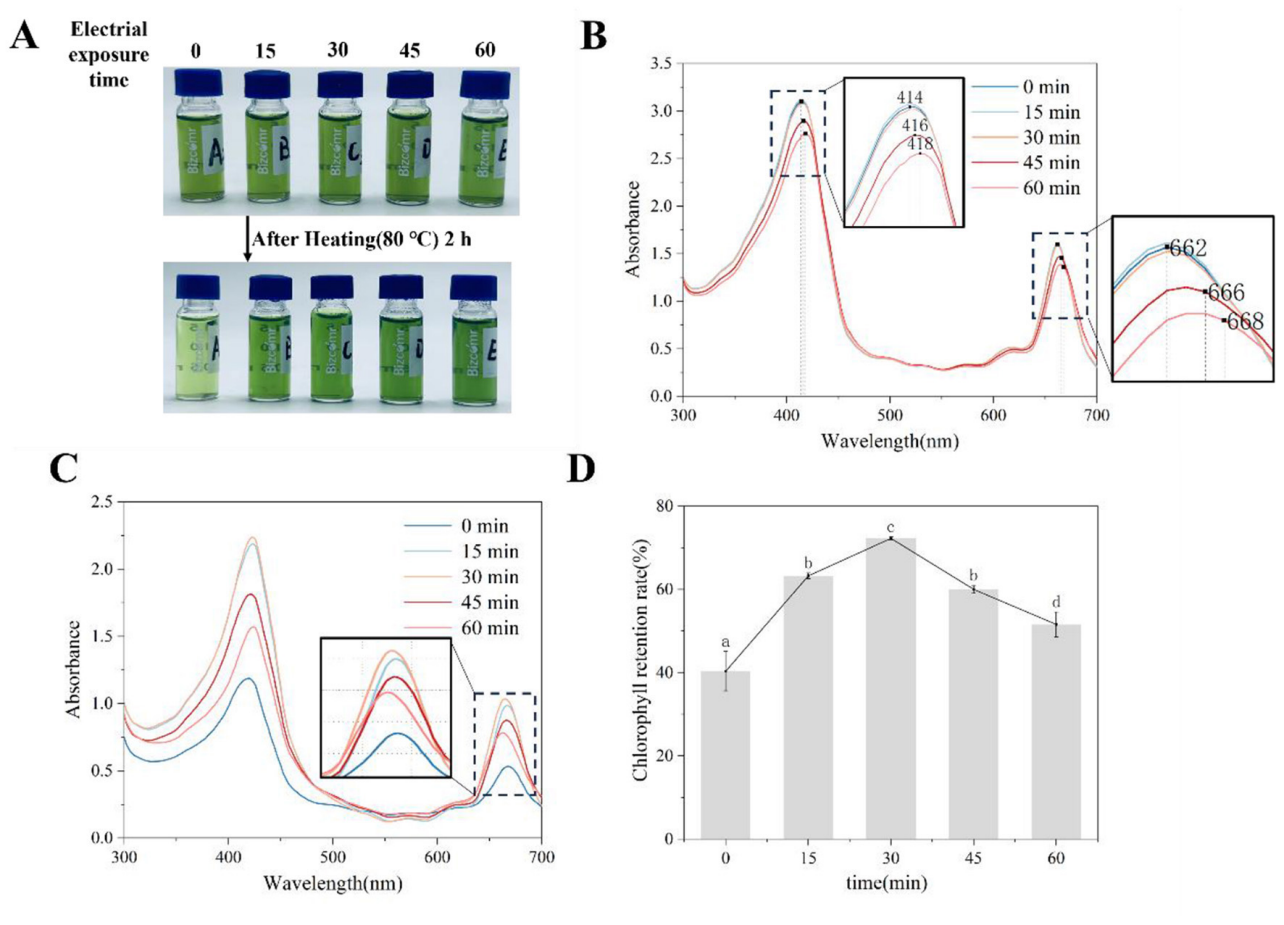
Effect of electric induction aggregation on the thermal stability of Chl: solution state **(A)**; UV absorption spectrum **(B)** after electric induction; UV absorption spectrum **(C)** and retention rate of Chl **(D)** after heating.

The results in Figure 2A show the UV absorption spectra recorded every 7.5 min during the first 30 min of electric induction. The five curves are completely overlapping, indicating that electric induction had no impact on the position of the characteristic curve in 30 min, which is consistent with the results shown in Figure 3B. This means that electric induction did not alter the spectral properties of Chl nor lead to the formation of any new substances. Furthermore, the overlapping spectra also confirm that no significant changes in concentration occurred during the first 30 min, implying that Chl did not form macroscopically visible precipitates, as shown in Figure 1.

The results in Figure 2B show the fluorescence emission spectra at a wavelength of 436 nm, recorded every 7.5 min during the first 30 min of electric induction. The emission peak observed at 674 nm is attributed to Chl a (31). As electric induction time increases, a significant decrease in fluorescence intensity is observed. Under the condition of constant Chl concentration, the occurrence of this fluorescence quenching phenomenon indicates that electric induction enhances intermolecular interactions, leading to aggregation between Chl molecules. Figure 2C shows the Chl concentration retention rate during 80 min of electric induction. Initially, there is no significant difference in the Chl concentration retention rate between 0 min and 20 min, which is consistent with the results shown in Figure 2A, where no change in concentration was observed during the first 30 min of electric induction. However, with prolonged induction time, significant differences in the Chl concentration retention rate are observed at each 20 min interval during the subsequent 60 min. This indicates that between 20 and 40 min of induction, Chl begins to form visible precipitates and aggregates, leading to a decrease in the Chl concentration in the solution. As the induction time increases, the Chl concentration in the solution continues to decline, meaning that increasing amounts of Chl precipitate. Since the decrease in Chl concentration retention rate is directly caused by aggregation and precipitation, this curve indirectly reflects the increase in aggregate amount over time under the specific voltage condition (1.5 V).

Figure 2D shows the changes in the UV absorption spectra of the Chl recorded every 20 min during 80 min of electric induction. Initially, the spectra for 0 min and 20 min overlap completely. After 20 min, the absorbance gradually decreases, confirming the continuous decline in Chl concentration.

In Figure 2B at the 15 min, a slight red shift is observed, which the peak wavelength shifts from 674 nm to 676 nm. In Figure 2D, as the duration of electric induction increases, a more pronounced red shift is observed in the UV absorption spectra of Chl. The Chl subjected to different durations of electric induction exhibits a soret band absorption peak between 400 and 500 nm, along with Q_x_ band absorption peaks between 500 and 600 nm and a prominent Q_y_ band absorption peak around 670 nm. The results confirm that the spectra of Chl after electric induction align with the characteristic UV absorption profile of standard Chl (32). Additionally, no new peaks were observed with the extension of the electric induction, confirming that no other substances were produced. With the extension of the induction time, a slight red shift in the soret band absorption peak and the Q_y_ band absorption peak was observed at 40 min. By 80 min, this red shift became more pronounced for both peaks. It should be noted that fluorescence intensity reflects the excited-state properties of the porphyrin ring, not the Chl concentration. When Chl molecules approach each other, intermolecular interactions (charge transfer or energy transfer) deactivate the excited state, leading to fluorescence quenching. In contrast, UV-Vis absorption is directly correlated with Chl concentration. Therefore, the fluorescence quenching observed within 30 min (Figure 2B) indicates that Chl molecules are approaching each other, while the unchanged UV-Vis spectra (Figure 2A) confirm that the Chl concentration remains constant during this period. These two results are not contradictory. They reveal that molecular proximity (detected by fluorescence) precedes precipitation (detected by UV-Vis concentration decrease). The significant red shift and concentration decrease observed after 40 min (Figure 2D) mark the transition to larger aggregates and the onset of precipitation. Chl is a unique porphyrin molecule, and the highly conjugated structure of porphyrin facilitates the formation of J- and H-type aggregates (33). Typically, for most porphyrin aggregates, the aggregation pattern is not purely J- and H-type aggregates. Under certain conditions, J- and H-type aggregates can either coexist or transition between forms depending on the environment (34). The observed red shift phenomenon corresponds to the significant red shift effect typically seen in J-type aggregation of Chl (15). In contrast, H-type aggregation is named after the hypochromic shift, this type of aggregate shows a distinct blue shift in its characteristic absorption peak compared to its monomer (35). As a result, the observed red shift in the UV-Vis absorption spectrum (Figure 2D) is characteristic of J-type aggregation, which features a head-to-tail molecular arrangement. This suggests that the Chl aggregates formed by electric induction exhibit J-type aggregation behavior.

**Figure 4:**
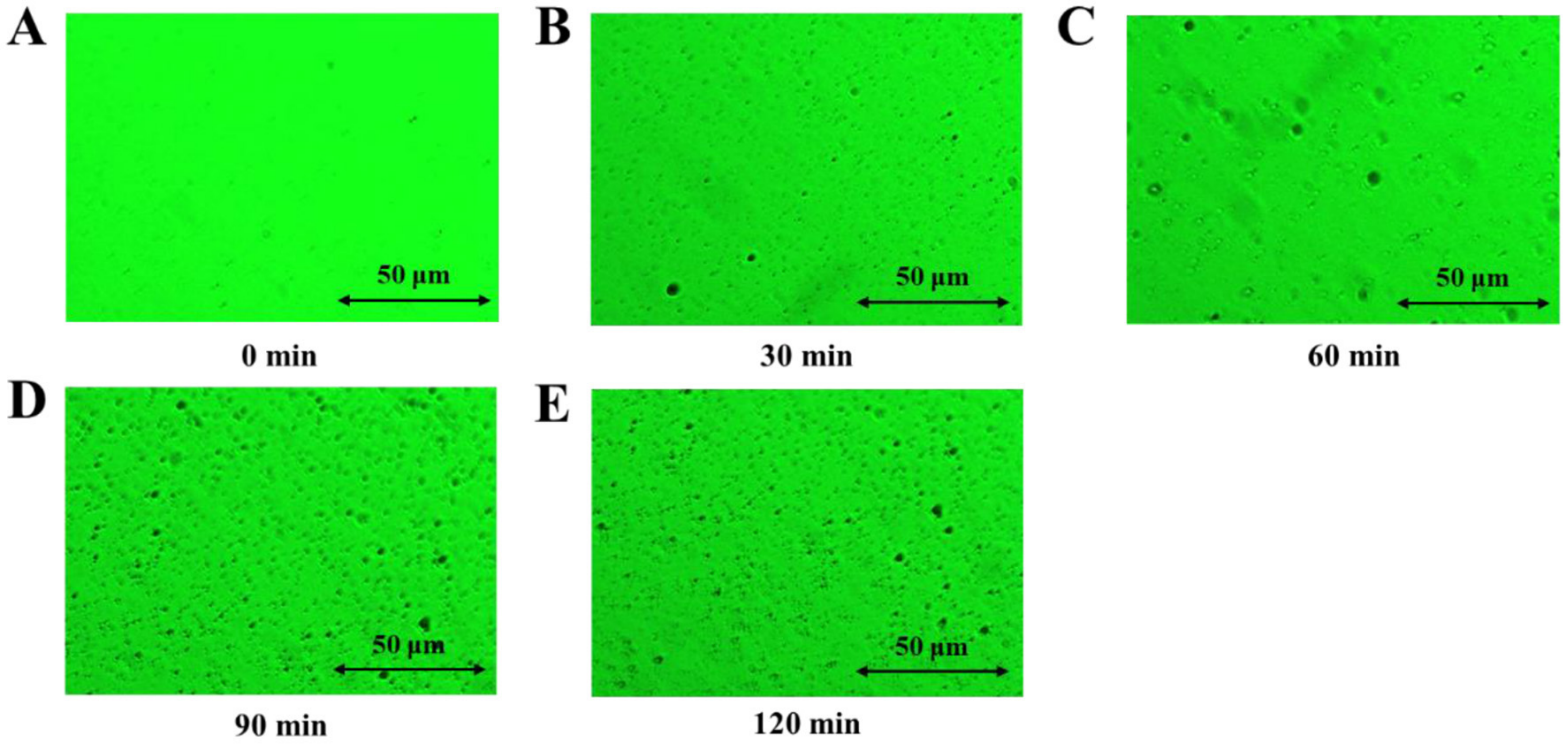
The inverted fluorescence microscopy results of the Chl from 0 to 120 min (**A**-**E**).

Figure 4 presents the Chl observed under an inverted fluorescence microscope at 0 min, 30 min, 60 min, 90 min, and 120 min of electric induction. Compared to Figures 4B–E, Figure 4A shows a uniform fluorescence distribution of Chl across the field of view, with no observable aggregated particles. This indicates that Chl is uniformly dispersed in very small sizes. In Figure 4B, after 30 min of electric induction, a small number of large aggregates begin to appear. As the electric induction time increases, the number of aggregates grows, demonstrating that induction brings Chl molecules closer together, promoting the formation of self-aggregates.

Consequently, due to electric induction, the distance between Chl molecules shortens, resulting in intermolecular forces that prompt aggregation. The red shift observed in the UV-Vis absorption spectrum (Figure 2D) is characteristic of J-type aggregation (an end-to-end or head-to-tail molecular arrangement), suggesting that the formed aggregates exhibit J-type-like behavior. However, we acknowledge that definitive confirmation of J-aggregate formation would require additional techniques. As the electric induction duration elongates, these aggregates progressively expand and multiply, ultimately yielding visible precipitates. In subsequent experiments, we will investigate the aggregation tendency of Chl under electric induction and the intermolecular interactions that contribute to the formation of aggregates.

### 3.2 Investigation of Chl aggregation tendencies and forms under electric induction

**Figure 5:**
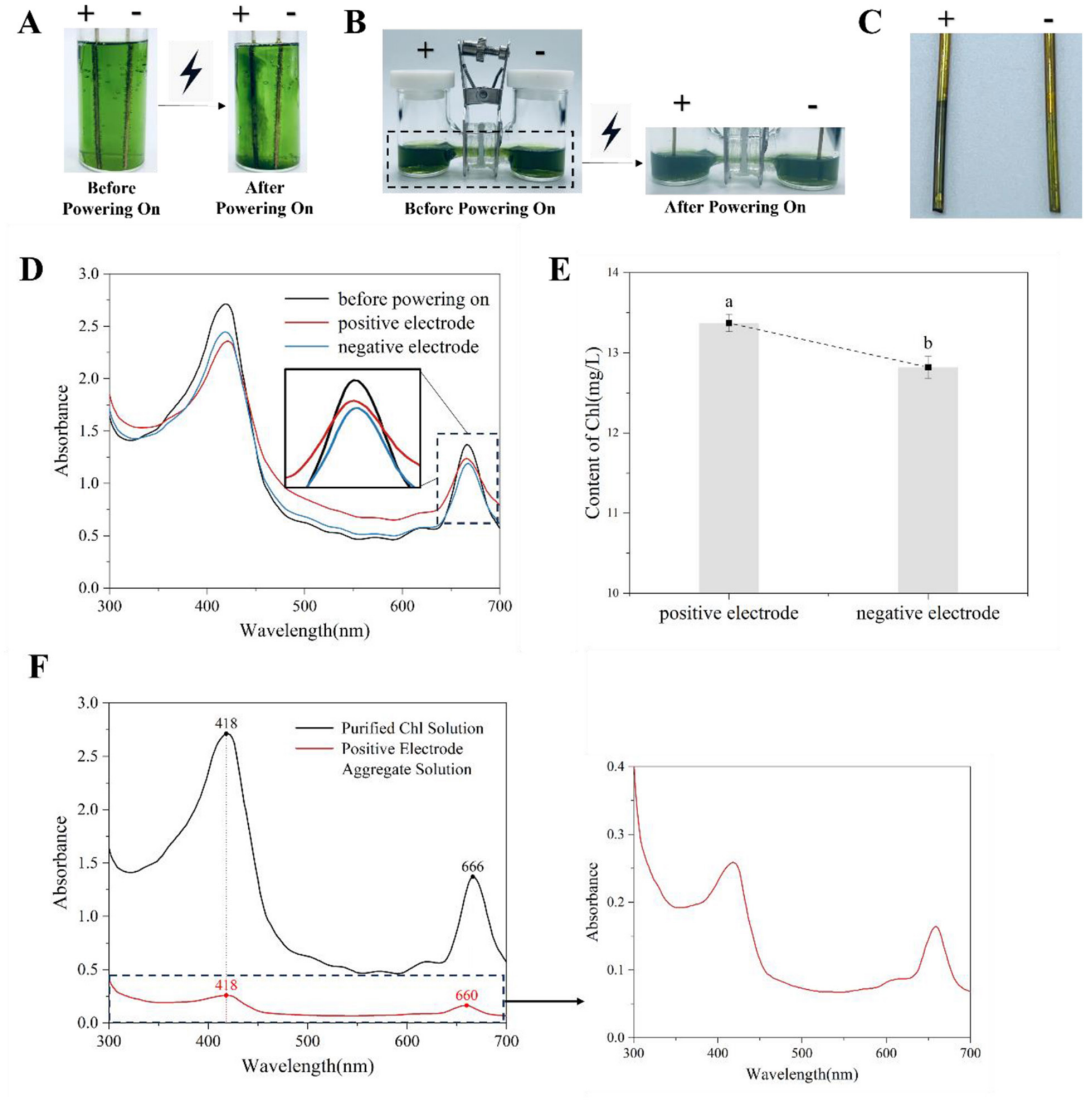
Aggregation direction of Chl with 2 h electric induction: Chl solution **(A)** and which in a specialized device **(B)**; the positive and negative electrodes **(C)**; UV absorption spectra of the Chl **(D)**; Chl concentration **(E)**; UV absorption spectrum of the samples from the positive electrode **(F)**.

During the electric induction process of Chl, the result was illustrated in Figure 5A, where precipitates formed at the positive electrode and continued to increase in quantity. Therefore, it is suggested that under the applied electric field, Chl molecules migrate toward the positive electrode due to their polarity, leading to their accumulation and subsequent aggregation with increasing induction time. To demonstrate this phenomenon, a specialized device shown in the Supplementary Figure S1 was utilized. It could create a greater separation (3 cm) between the positive and negative electrode, ensuring that electric induction affects the Chl while facilitating the measurement of differences in solution properties between the two electrodes. After 2 h of electric induction, it was illustrated from Figure 5B that Chl at the positive electrode appears more turbid. Samples of the solutions from both positive and negative electrode before and after electric induction were collected for UV absorption spectroscopy analysis, with the results presented in Figure 5D. On one hand, the UV absorption spectra from both electrode regions exhibited an overall decrease after induction, indicating that the electric field itself promoted Chl aggregation and precipitation, thereby reducing the Chl concentration in the bulk solution. On the other hand, after 2 h of induction, the UV absorption spectrum between 600 and 700 nm near the positive electrode was significantly higher than the negative electrode. As shown in Figure 5E, the Chl concentration at the positive electrode was significantly higher than the negative electrode. After the electric induction, both electrodes were taken out. As illustrated in Figure 5C, a distinct green deposit (the visible precipitate in Figure 1) was observed on the positive electrode compared to the negative. To verify the identity of this precipitate, the green deposit was washed off the positive electrode with ethanol and subjected to UV absorption spectrum analysis. The resulting UV absorption spectrum, shown in Figure 5F, exhibits a soret band absorption peak between 400 and 500 nm and a distinct Q_y_ band peak around 670 nm, consistent with the spectral characteristics of Chl (31). This spectral match confirms that the precipitate is composed of Chl. Since no other external substances were introduced in the experimental system, this confirms that the green deposit on the positive electrode was Chl aggregates.

**Figure 6:**
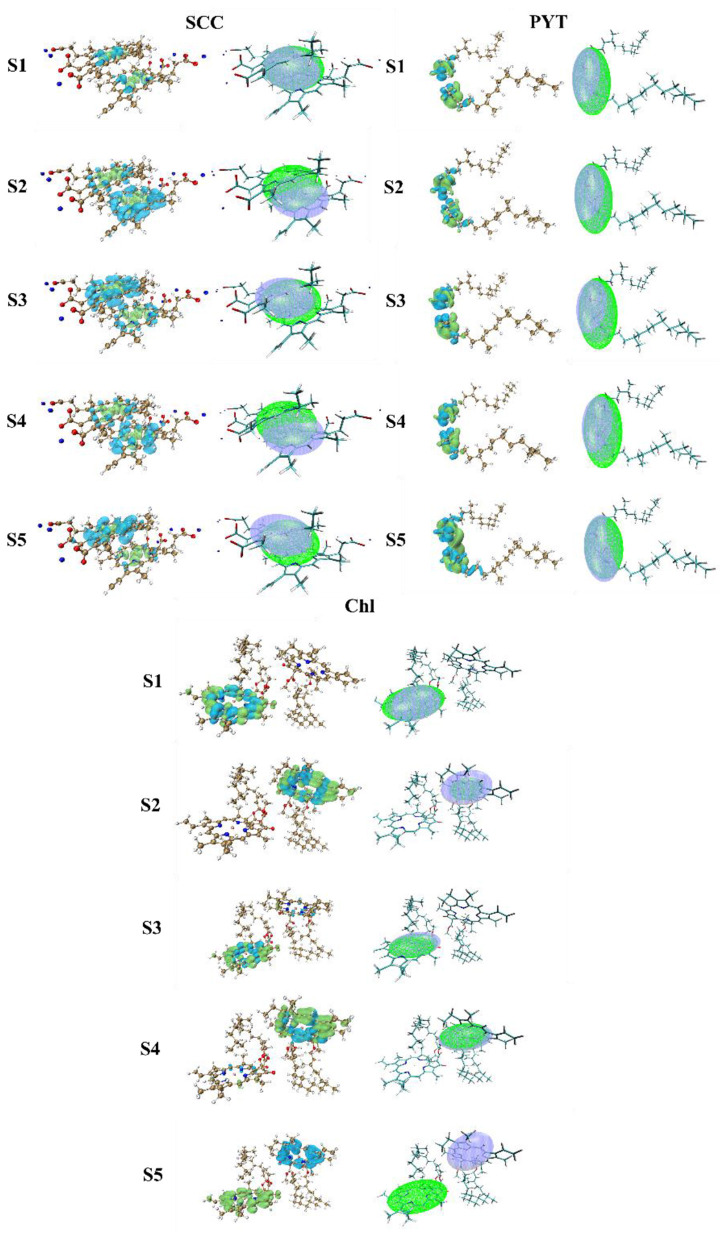
Hole electron analysis in SCC PYT and Chl dimer.

**Figure 7:**
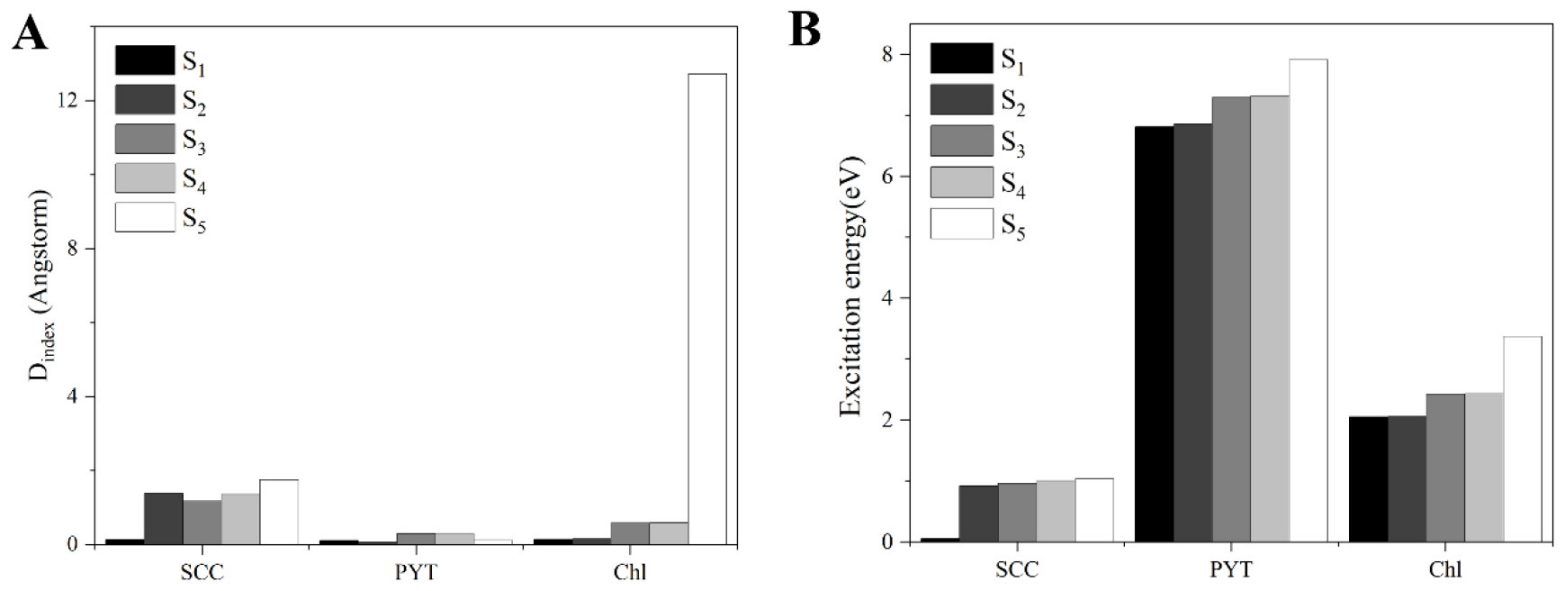
Centroid shift distance (D_index_) between hole and electron distribution regions **(A)** and excitation energies **(B)** of SCC, PYT and Chl.

Based on the experimental observations and quantum chemical calculations, the mechanism is proposed as follows. During electric induction, Chl molecules migrate toward the positive electrode due to their polarity under the applied electric field, generating a concentration gradient between the positive and negative electrode. As the induction time increases, the distance between Chl molecules at the positive electrode gradually decreases. Crucially, the conjugated porphyrin ring enables intermolecular charge transfer (CT) between adjacent molecules (Figures 6, 7), which stabilizes the aggregated state and promotes ordered assembly. These aggregates continue to grow with extended induction time, ultimately becoming visible precipitates at the positive electrode.

The fluorescence properties of Chl originate from its porphyrin ring structure and the rigid planar structure of this porphyrin ring promotes aggregation among Chl molecules (36). This inherent property, combined with electric-field-driven migration and CT, explains why Chl readily forms ordered aggregates under electric induction. The observed red shift in the UV-Vis absorption spectrum and fluorescence quenching are attributable to energy transfer between adjacent Chl molecules, resulting from changes in the relative spatial orientation of their porphyrin rings. These spectroscopic changes serve as evidence for the occurrence of Chl aggregation. Therefore, it is speculated that the highly conjugated structure of the porphyrin ring serves a more substantial function in electric induction compared to the phytyl tail of Chl.

To demonstrate that the electric induction effect on the porphyrin ring is more pronounced than on the phytyl tail, two experimental reagents were selected: sodium copper chlorophyllin (SCC) and phytol (PYT). SCC is a derivative of Chl produced through hydrolysis and copper substitution. After hydrolysis, the phytyl tail is removed, and with copper substitution the Mg^2^? in the porphyrin ring is replaced by Cu^2^?. Its main structure is essentially the porphyrin ring of Chl, with the primary difference being the metal ion in the porphyrin ring's center (37). Phytol (PYT; 3,7,11,15-tetramethyl-hexadec-2-en-1-ol), a compound widely present in nature, is part of the Chl molecule and primarily represents the phytyl tail structure of Chl (38).

**Figure 8:**
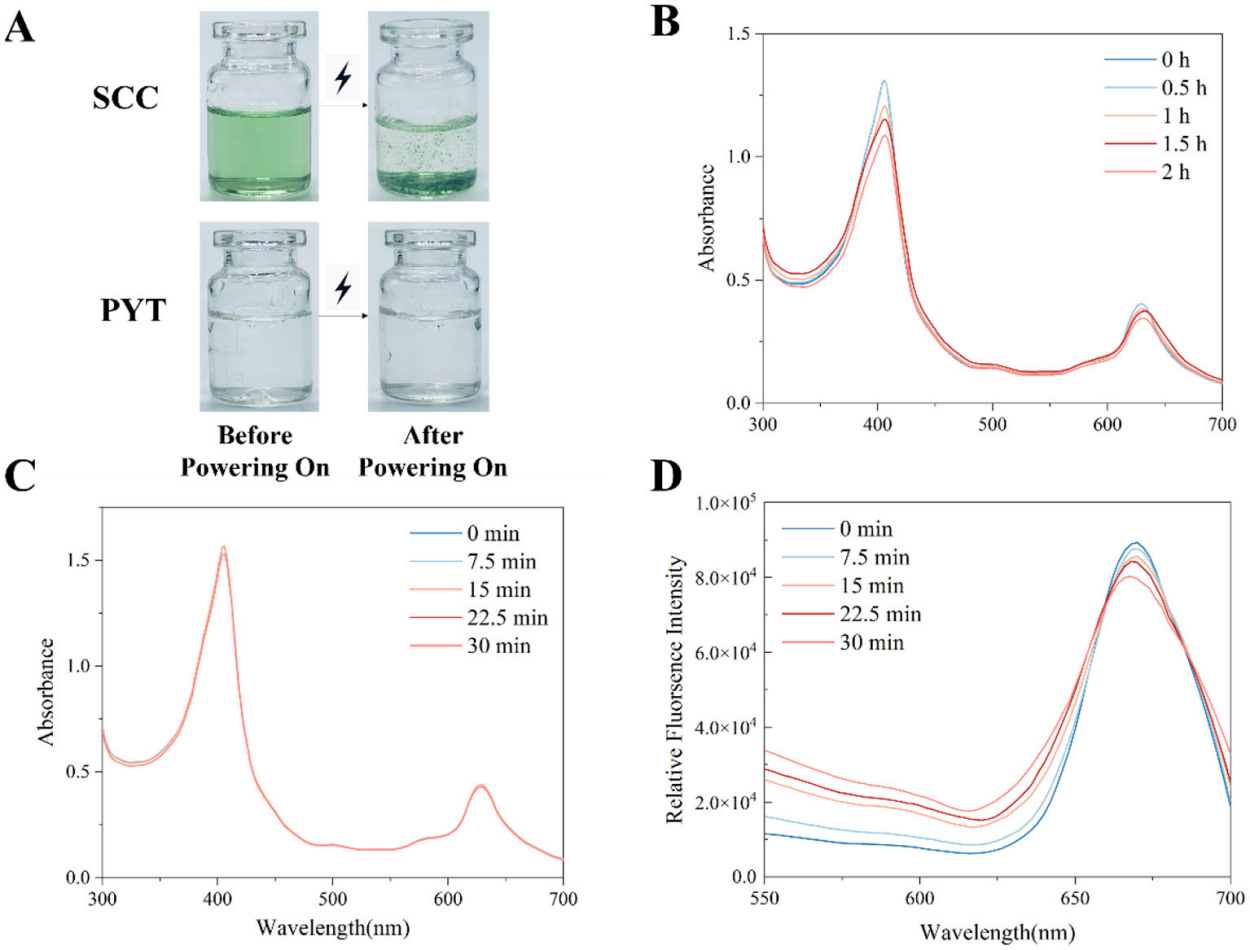
Effect of electric induction on Chl SCC and PYT: SCC and PYT after 24 h of electric induction **(A)**; UV absorption spectra of SCC with 2 h of electric induction **(B)**; UV absorption spectra of SCC with 30 min of electric induction **(C)**; fluorescence emission spectra of SCC with 30 min of electric induction **(D)**.

Figure 8A shows the changes in SCC and PYT after 24 h of electric induction. SCC produced a significant amount of precipitate, while PYT showed no notable change. This stark contrast demonstrates that the presence of the porphyrin ring is essential for electric-field-induced aggregation, as SCC (retaining the porphyrin ring) aggregated, whereas PYT (lacking the porphyrin ring) did not. Figure 8B presents the changes in the UV absorption spectra of SCC after 2 h of electric induction. No significant changes occurred within the first 30 min, but the spectra showed a gradual decline every subsequent 30 min, confirming that SCC began precipitating after 30 min, resulting in a decrease in concentration. Figure 8C displays the UV absorption spectra of SCC within the first 30 min, with spectra taken every 7.5 min. The five curves overlapped, ruling out concentration changes, structural alterations, or interference from other substances. Figure 8D presents the fluorescence emission spectra of SCC under excitation at 402 nm over 30 min (excitation wavelength determined as shown in the Supplementary Figure S2), revealing a trend of fluorescence quenching similar to the phenomenon Chl with electric induction. This indicates that as Chl, SCC aggregated under electric induction within 0.5 h, and the aggregation increased over time, leading to precipitation. The close resemblance between SCC and Chl aggregation behavior confirms that the porphyrin ring (not the phytyl tail) is the key structural motif responsible for electric-field-induced aggregation. Given the contrasting behaviors of SCC (which retains the porphyrin ring) and PYT (which consists solely of the phytyl tail), it is confirmed that the promotion of Chl aggregation under electric induction is primarily due to the effect on the porphyrin ring. Thus, this structural comparison serves as a critical validation of the proposed mechanism: the porphyrin ring enables intermolecular charge transfer (CT) and drives aggregation.

Based on these findings, it is reasonable to speculate that other porphyrin-containing compounds—such as heme, metal-porphyrin complexes, and other chlorophyll derivatives (e.g., pheophytin)—may also exhibit similar electric-field-induced aggregation behavior. This principle could guide the selection of candidate compounds for future studies on electric-field-assisted extraction and separation.

CT generally refers to an interaction between a charge donor and an acceptor. When the two are close to each other, the empty orbit of the charge acceptor overlaps with the electron cloud of the charge donor, producing a CT interaction. To elucidate the molecular mechanism underlying electric-field-induced aggregation, particularly the role of the porphyrin ring in enabling intermolecular interactions, excited-state calculations for the dimers of SCC, PYT, and Chl were performed as shown in Figure 6. It shows the hole-electron analysis of SCC, PYT, and Chl. For each material, the left-side images of each row show the distribution zones before and after electron excitation, while the right-side images focus on the center of mass and the extent of the distribution areas in both conditions. The blue regions indicate the positions where the electron has departed, creating a hole, while the green regions mark the locations where the electron has been transferred.

From the SCC of Figure 6, in the S1, there is significant overlap between the hole and electron regions with electrons evenly distributed across the porphyrin rings of both molecules before and after excitation, indicating a localized excitation. However, in the left-side diagram of the SCC, starting from the S2, the hole and electron regions become distinctly separated, indicating the occurrence of intermolecular CT, though some overlap remains. This suggests that in the S2–S5 excited states of SCC, both intermolecular CT and intramolecular localized excitation coexist. This coexistence indicates that SCC readily supports intermolecular CT upon excitation, which is a prerequisite for electric-field-induced aggregation.

In the PYT of Figure 6, the electron distribution before and after excitation, from S1 to S5, is relatively uniform across the head groups of both molecules. On the other hand, in the Chl of Figure 6, the electron distribution in the S1 to S4 states remains localized on one porphyrin ring of the dimer, while in the S5 state, the electron distribution shifts completely from one porphyrin ring to the other upon excitation.

The D_index_ reflects the centroid movement distance between the hole region and the electron region in the excited state. In Figure 7A, the D_index_ for SCC in the S1 is 0.143 Å, while in the S2, the D_index_ increases to 1.382 Å. Since the bond length of a typical carbon-carbon single bond is around 1.54 Å, the small D_index_ value in the S1 state indicates minimal centroid movement, suggesting that the excitation mainly occurs within a single molecule, classified as localized excitation. In contrast, the D_index_ in the S2–S5 are significantly larger than the S1, indicating increased centroid movement and further supporting the idea that excitation in these states involves more intermolecular interaction rather than localized excitation. Additionally, the D_index_ for PYT in different excited states, shown in Figure 7A are: S1 = 0.113 Å, S2 = 0.063 Å, S3 = 0.300 Å, S4 = 0.306 Å, and S5 = 0.117 Å. All these values are relatively low, showing minimal changes across the different states. Coupled with the molecular orbital diagrams where no significant differences in electron distribution were observed, this confirms that no intermolecular CT occurs in the S1–S5, and all excitations remain localized within the molecule. Since PYT lacks the porphyrin ring, this result demonstrates that the porphyrin ring is essential for enabling intermolecular CT. For Chl, as shown by the D_index_ in Figure 7A, the dimer exhibits the following values: S1 = 0.150 Å, S2 = 0.155 Å, S3 = 0.583 Å, S4 = 0.591 Å, and S5 = 12.725 Å. The D_index_ remains low from S1 to S4, confirming localized excitation in these states. However, the significant increase in the D_index_ in the S5, which reaches 12.725 Å, indicates substantial separation between the hole and electron regions, confirming that intermolecular CT occurs in the S5. This CT interaction, occurring when Chl molecules are sufficiently close, provides the stabilizing force for aggregate formation under electric induction.

Figure 7B presents the excitation energies of SCC, PYT and Chl across different excited states. For SCC, the excitation energy in the S2 is 0.919 eV, which is significantly higher than the 0.050 eV observed in the S1. This notable increase in excitation energy suggests that CT begins from the S2 state. Additionally, for Chl, the excitation energy in the S5 is markedly higher than in the other four states, further confirming that intermolecular CT occurs specifically in the S5.

Comparing the three substances, the excitation energy of PYT in all excited states is notably higher than SCC and Chl, indicating that PYT requires significantly more energy to achieve excited state. This explains why under electric induction, PYT does not exhibit substantial changes, consistent with the experimental observation that after 24 h of electric induction, SCC produced a visible precipitate, while PYT did not have significant changes.

In Figure 7B, SCC excitation energy is clearly lower than that of Chl, meaning SCC experiences intermolecular CT more easily. This computational result is consistent with the experimental observation that SCC precipitated more readily than Chl under electric induction (Figure 8A), further validating that CT efficiency directly correlates with aggregation propensity. The main structural differences between SCC and Chl is the central metal ion and the phytyl tail. The structure of the phytyl tail closely resembles that of PYT, and from the quantum chemical simulations of PYT, it is evident that intermolecular CT is difficult to achieve. Therefore, when the phytyl tail is removed, the energy required for CT in SCC is significantly reduced. The Cu^2+^ in SCC, due to its strong electrostatic interaction, interferes with charge movement. When an electron departs from Cu^2+^ on the porphyrin ring, the Cu^2+^ inhibits its movement. Conversely, when the electron approaches aCu^2+^ on a neighboring porphyrin ring, this Cu^2+^ facilitates the electron's movement, and finally enhance CT. This results in charge movements within SCC occurring both between the porphyrin rings of two molecules and between Cu^2+^ and N of the same or different porphyrin rings. Thus, we hypothesize that if the phytyl tail and the central metal ion were removed, the resulting Chl derivative might exhibit a much higher CT efficiency compared to Chl. In summary, the quantum chemical calculations reveal that: (i) intermolecular CT occurs in Chl at the S5 excited state (D_index_ = 12.725 Å), providing the driving force for aggregation; (ii) PYT, lacking the porphyrin ring, exhibits no CT across all excited states, confirming the essential role of the porphyrin ring; and (iii) SCC, which lacks the phytyl tail, exhibits CT at a lower excitation energy (S2, 0.919 eV) than Chl, explaining its higher aggregation propensity. These results collectively elucidate the molecular mechanism: the electric field brings Chl molecules into close proximity, whereupon the porphyrin ring enables intermolecular CT, which stabilizes the aggregated state and drives the formation of ordered assemblies. The consistency between the quantum chemical calculations and experimental observations—particularly that SCC precipitates more readily than PYT—further validates this mechanism.

It should be noted that while the quantum chemical calculations support intermolecular charge transfer (CT) between Chl molecules, which is a prerequisite for ordered aggregate formation, direct structural confirmation of J-aggregates was not performed in this study. The assignment of J-type aggregation is therefore primarily based on the characteristic red shift in the UV-Vis absorption spectrum and the fluorescence quenching behavior (Figure 2), which are well-established indicators of J-type aggregation in the literature (15).

**Figure 9:**
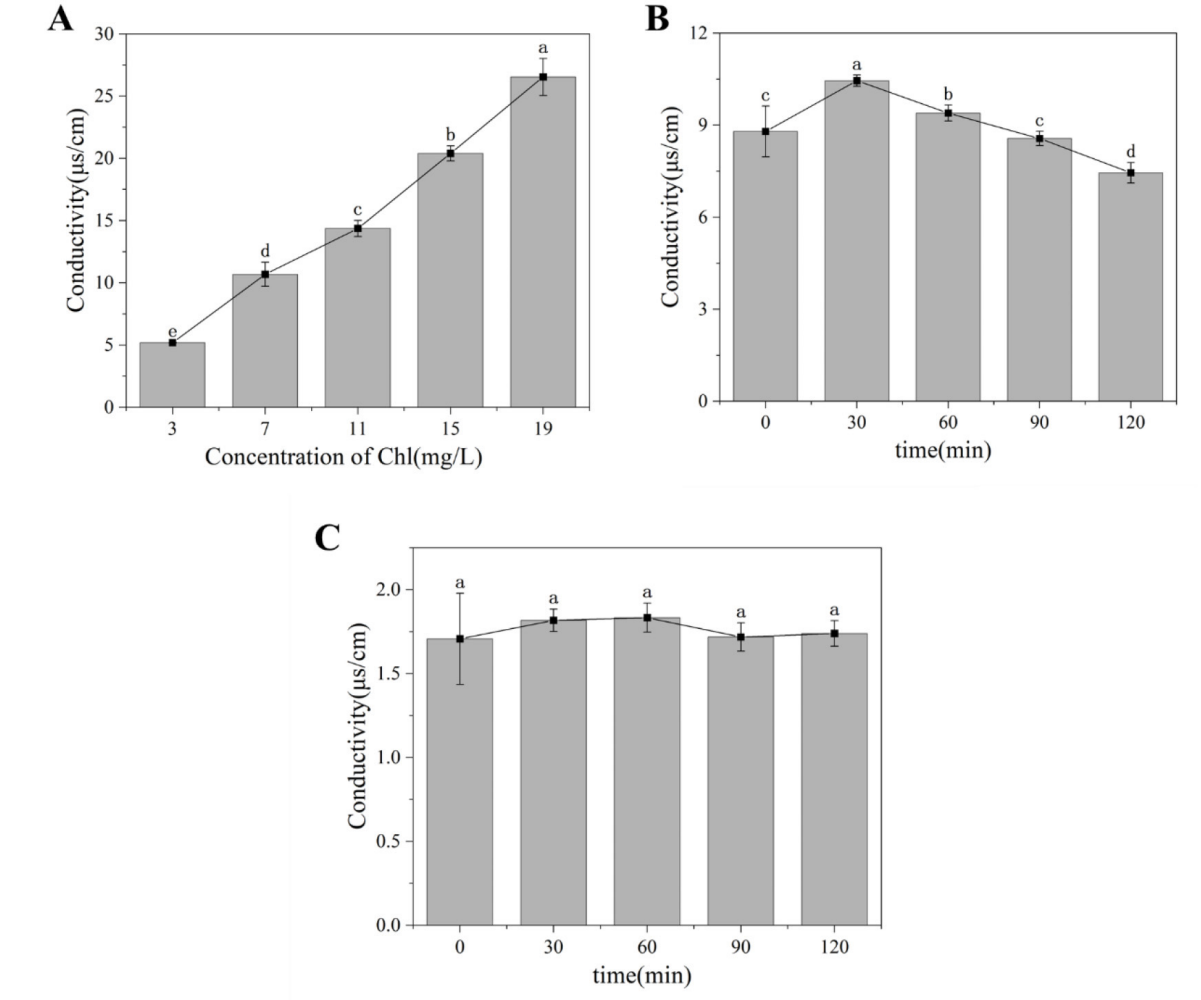
Effect of Chl concentration **(A)** and electric induction time **(B)** on the conductivity of Chl solution. Conductivity changes of ethanol-water solution within electric induction **(C)**.

Conductivity is directly related to CT, as both reflect the electron transfer process. The more active the CT bonds within a sample, the higher its conductivity. Figure 9A shows that as Chl concentration increases, conductivity also rises. The increase in Chl concentration implies more self-aggregation, which leads to enhanced conductivity, consistent with the research of Li et al. (16). In Figure 9B, conductivity increases during the first 30 min of electrical induction. At 60 min, despite a decrease in concentration, the conductivity remains significantly higher than at 0 min, although lower than at 30 min. This is because the continuous formation of Chl self-aggregates causes the conductivity to remain elevated even as the concentration decreases. The decrease in conductivity from 30 to 120 min is attributed to the growth and precipitation of Chl aggregates, reducing the concentration of dispersed Chl in the solution.

To exclude the possibility that the observed conductivity changes were due to electrolytically generated ions, a control experiment was performed. As shown in Figure 9C, the pure ethanol/water solvent (without Chl) showed no significant change in conductivity after electric induction, indicating that under this experimental conditions (1.5 V, ethanol/water system), no substantial ions were generated from the solvent or the copper electrodes. Since the pure solvent produced no conductivity increase, the conductivity increase observed in the Chl solution (Figure 9B) cannot be attributed to ions. Conversely, because the Chl solution exhibited a clear conductivity increase during aggregation while the pure solvent did not, the enhanced conductivity must originate from the Chl aggregates themselves.

Therefore, the enhanced conductivity is attributed specifically to CT between porphyrin rings within the Chl aggregates, not to free ions in solution. This conclusion macroscopically confirms the quantum chemical calculations, which predicted intermolecular CT between Chl molecules. The aggregation occurs in a low-ionicity solvent system, and the electrical signal accompanying aggregation originates from intramolecular CT within the porphyrin rings, directly addressing the concern that ions might mediate the aggregation process.

In this study, a fixed voltage of 1.5 V and copper electrodes were used to establish the fundamental phenomenon of electric-field-induced Chl aggregation. We acknowledge that both voltage intensity and electrode material may influence aggregation efficiency, aggregate morphology, and precipitation kinetics. Systematic investigations of different voltages and electrode materials (e.g., platinum, graphite, or stainless steel) will be conducted in future work to optimize the extraction and stabilization process.

### 3.3 Effect of electrical induction aggregation to the stability of Chl

Chl self-aggregation has been shown to improve the thermal stability of Chl. Based on the observation that electrical induction promotes Chl molecular aggregation, it is hypothesized that electrical induction aggregation also enhances the thermal stability of Chl solutions. Five groups of 15 mg/L Chl ethanol/water (v/v = 1:1) solutions were prepared with different electrical induction times. After induction, the solutions were heated at 80 °C for 2 h, and the experimental results are shown in Figure 3. Figure 3A displays photos of the solutions after heating. Notably, the 0 min group showed significant fading after heating, while the other four groups exhibited no visible changes. Figure 3B presents the UV absorption spectra after electrical induction, consistent with the results obtained in Section 3.1: the spectra of the 0 min, 15 min, and 30 min groups showed no significant changes, while the 45 min group exhibited a red shift due to the formation of aggregation, leading to a decrease in concentration. Figure 3C shows the changes in the UV absorption spectra of the solutions after heating. The curve of the 0 min group after heating was significantly lower than those of the other four electrically induced groups. Figure 3D displays the Chl retention rates after heating for 2 h at 80 °C with different induction times. “0 min” denotes the control group, meaning the Chl solution without any electric field induction treatment. The average retention rate of Chl in the control group was 39.50%, while the retention rates for the 15 min, 30 min, 45 min, and 60 min groups were 62.81%, 70.48%, 59.14%, and 52.47%, respectively. The highest retention rate was observed in the 30 min group, which improved by 78.43% compared to the control group. This confirms that electrical induction promotes Chl self-aggregation, significantly enhancing the thermal stability of Chl. However, the 45 min and 60 min groups exhibited a decrease in thermal stability compared to the 15 min and 30 min groups, due to the precipitation of Chl after 30 min of induction, leading to a reduction in concentration, and consequently a reduction of thermal stability. In the first 30 min of electrical induction, before precipitation occurs, longer induction times resulted in increased Chl self-aggregation and improved thermal stability.

In conclusion, the electrical induction aggregation process can be divided into two stages. During the first 30 min of induction, Chl form aggregates through self-assembly without precipitation. These aggregates significantly enhance the thermal stability of Chl, making this stage suitable for improving Chl thermal stability. After 45 min or longer, Chl begin to precipitate, causing a decrease in Chl concentration. While the thermal stability of the aggregates decreases at this stage, the precipitation facilitates the separation of Chl from other common pigments in spirulina, such as β-carotene and lutein, which supports a better approach for Chl extraction.

## 4 Conclusion

In summary, this study introduces a new approach for inducing Chl aggregation through electrical induction. The mechanism lies in the highly conjugated structure of the Chl porphyrin ring, which enables intermolecular CT under electric induction, leading to aggregate formation. As induction time increases, the aggregates grow larger, eventually causing Chl to precipitate and facilitating its separation from other substances. This approach demonstrates a two-stage application potential: initially, the formation of stable aggregates enhances the thermal stability of Chl, while at later stages, the formation of precipitates allows for efficient separation. Conventional electrocoagulation is a broad-spectrum impurity removal process that non-selectively captures various pollutants. In contrast, the proposed method induces selective self-aggregation of Chl under an electric field, with the ultimate goal of achieving directional extraction of Chl from complex mixtures. Future work will focus on achieving selective separation of Chl from impurities using electric field dissociation technology. This electric induction-based approach reduces organic solvent consumption, operates under mild conditions, does not alter the molecular structure of Chl, and avoids the side effects caused by the introduction of other substances, offering a more sustainable alternative to conventional extraction methods. The reduction in extraction costs and process complexity will promote the widespread application of Chl in industries such as food, cosmetics, and pharmaceuticals, creating new opportunities for the development and utilization of natural pigments.

## Data Availability

The original contributions presented in the study are included in the article/Supplementary material, further inquiries can be directed to the corresponding authors.
