# Supplementary material for: Electric-field-induced self-assembly of chlorophyll enables extraction and stabilization
Source: Front Nutr. 2026 Jul 7;13:1844456. doi: 10.3389/fnut.2026.1844456 (PMC13385226; doi:10.3389/fnut.2026.1844456)
Supplement: Supplementary file 1 [file Table_1.docx]

Supporting Information

**Electric-Field-Induced Self-Assembly of Chlorophyll Enables Extraction and Stabilization**

Yangbin Wang^1†^, Jingzhi Xue^1†^, Shuyu Wang^1^, Yixiao Liu^1^, Fangwei Li^1^[[1]](#footnote-1)^*^, Mingyong Zeng^1^[[2]](#footnote-2)^*^

^1^ State Key Laboratory of Marine Food Processing and Safety Control, College of Food Science and Engineering, Ocean University of China, Qingdao 266404, China/Sanya Institute of Oceanography, Ocean University of China, Sanya 572000, China.

**Table of Contents**

[1.Tables 3](#_Toc181208103)

[2.Figures 8](#_Toc181208104)

1. Tables

**Table S1** The orbital contributions of S1-S5 in SCC a dimer (contribution rate>1.0%).

| Excited states | Orbitals | Number of occupied electrons | Contribution rates to holes | Contribution rates to electrons |
| --- | --- | --- | --- | --- |
| S1 | 371（HOMO） | 2 | 100.253 % | 0.000 % |
|  | 372（LUMO） | 0 | 0.000 % | 99.999 % |
| S2 | 367 | 2 | 2.334 % | 0.000 % |
|  | 369 | 2 | 11.130 % | 0.000 % |
|  | 370 | 2 | 85.928 % | 0.000 % |
|  | 372 | 0 | 0.000 % | 100.431 % |
| S3 | 367 | 2 | 1.432 % | 0.000 % |
|  | 368 | 2 | 15.455 % | 0.000 % |
|  | 369 | 2 | 75.017 % | 0.000 % |
|  | 370 | 2 | 7.170 % | 0.000 % |
|  | 372 | 0 | 0.000 % | 100.339 % |
| S4 | 367 | 2 | 55.038 % | 0.000 % |
|  | 368 | 2 | 28.750 % | 0.000 % |
|  | 369 | 2 | 7.440 % | 0.000 % |
|  | 370 | 2 | 6.737 % | 0.000 % |
|  | 372 | 0 | 0.000 % | 100.296 % |
| S5 | 367 | 2 | 38.715 % | 0.000 % |
|  | 368 | 2 | 53.266 % | 0.000 % |
|  | 369 | 2 | 6.162 % | 0.000 % |
|  | 372 | 0 | 0.000 % | 100.254 % |

**Table S2** The orbital contributions of S1-S5 in PYT dimer (contribution rate>1.0%).

| Excited states | Orbitals | | Number of occupied electrons | Contribution rates to holes | Contribution rates to electrons |
| --- | --- | --- | --- | --- | --- |
| S1 | 165 | 2 | | 3.446 % | 0.000 % |
|  | 166 | 2 | | 6.326 % | 0.000 % |
|  | 167 | 2 | | 30.813 % | 0.000 % |
|  | 168（HOMO） | 2 | | 56.757 % | 0.000 % |
|  | 169（LUMO） | 0 | | 0.000 % | 62.192 % |
|  | 170 | 0 | | 0.000 % | 34.224 % |
|  | 171 | 0 | | 0.000 % | 1.169 % |
| S2 | 165 | 2 | | 3.872 % | 0.000 % |
|  | 166 | 2 | | 3.201 % | 0.000 % |
|  | 167 | 2 | | 48.107 % | 0.000 % |
|  | 168（HOMO） | 2 | | 41.873 % | 0.000 % |
|  | 169（LUMO） | 0 | | 0.000 % | 52.359 % |
|  | 170 | 0 | | 0.000 % | 44.630 % |
| S3 | 163 | 2 | | 1.150 % | 0.000 % |
|  | 165 | 2 | | 27.832 % | 0.000 % |
|  | 166 | 2 | | 54.090 % | 0.000 % |
|  | 167 | 2 | | 8.279 % | 0.000 % |
|  | 168（HOMO） | 2 | | 3.792 % | 0.000 % |
|  | 169（LUMO） | 0 | | 0.000 % | 57.574 % |
|  | 170 | 0 | | 0.000 % | 37.521 % |
|  | 171 | 0 | | 0.000 % | 2.406 % |
| S4 | 163 | 2 | | 1.984 % | 0.000 % |
|  | 165 | 2 | | 45.159 % | 0.000 % |
|  | 166 | 2 | | 38.567 % | 0.000 % |
|  | 167 | 2 | | 6.016 % | 0.000 % |
|  | 168（HOMO） | 2 | | 1.868 % | 0.000 % |
|  | 169（LUMO） | 0 | | 0.000 % | 53.770 % |
|  | 170 | 0 | | 0.000 % | 42.703 % |
|  | 171 | 0 | | 0.000 % | 1.091 % |
| S5 | 141 | 2 | | 1.840 % | 0.000 % |
|  | 142 | 2 | | 1.057 % | 0.000 % |
|  | 149 | 2 | | 1.017 % | 0.000 % |
|  | 150 | 2 | | 2.245 % | 0.000 % |
|  | 160 | 2 | | 3.763 % | 0.000 % |
|  | 165 | 2 | | 9.691 % | 0.000 % |
|  | 166 | 2 | | 8.453 % | 0.000 % |
|  | 167 | 2 | | 27.851 % | 0.000 % |
|  | 168（HOMO） | 2 | | 36.105 % | 0.000 % |
|  | 169（LUMO） | 0 | | 0.000 % | 25.316 % |
|  | 170 | 0 | | 0.000 % | 40.692 % |
|  | 171 | 0 | | 0.000 % | 29.733 % |

**Table S3** The orbital contributions of S1-S5 in Chl dimer (contribution rate>1.0%).

| Excited states | Orbitals | Number of occupied electrons | | Contribution rates to holes | Contribution rates to electrons |
| --- | --- | --- | --- | --- | --- |
| S1 | 479 | | 2 | 10.456 % | 0.000 % |
|  | 481 | | 2 | 85.486 % | 0.000 % |
|  | 482（HOMO） | | 2 | 2.041 % | 0.000 % |
|  | 483（LUMO） | | 0 | 0.000 % | 85.522 % |
|  | 484 | | 0 | 0.000 % | 2.112 % |
|  | 485 | | 0 | 0.000 % | 9.918 % |
| S2 | 480 | | 2 | 10.581 % | 0.000 % |
|  | 481 | | 2 | 1.994 % | 0.000 % |
|  | 482（HOMO） | | 2 | 85.353 % | 0.000 % |
|  | 483（LUMO） | | 0 | 0.000 % | 2.115 % |
|  | 484 | | 0 | 0.000 % | 85.441 % |
|  | 486 | | 0 | 0.000 % | 9.989 % |
| S3 | 479 | | 2 | 64.917 % | 0.000 % |
|  | 480 | | 2 | 9.400 % | 0.000 % |
|  | 481 | | 2 | 20.688 % | 0.000 % |
|  | 482（HOMO） | | 2 | 2.770 % | 0.000 % |
|  | 483（LUMO） | | 0 | 0.000 % | 66.325 % |
|  | 484 | | 0 | 0.000 % | 9.341 % |
|  | 485 | | 0 | 0.000 % | 19.669 % |
|  | 486 | | 0 | 0.000 % | 2.687 % |
| S4 | 479 | | 2 | 9.168 % | 0.000 % |
|  | 480 | | 2 | 64.572 % | 0.000 % |
|  | 481 | | 2 | 2.982 % | 0.000 % |
|  | 482（HOMO） | | 2 | 21.048 % | 0.000 % |
|  | 483（LUMO） | | 0 | 0.000 % | 9.164 % |
|  | 484 | | 0 | 0.000 % | 66.046 % |
|  | 485 | | 0 | 0.000 % | 2.858 % |
|  | 486 | | 0 | 0.000 % | 19.979 % |
| S5 | 482（HOMO） | | 2 | 99.800 % | 0.000 % |
|  | 483（LUMO） | | 0 | 0.000 % | 99.444 % |

2. Figures


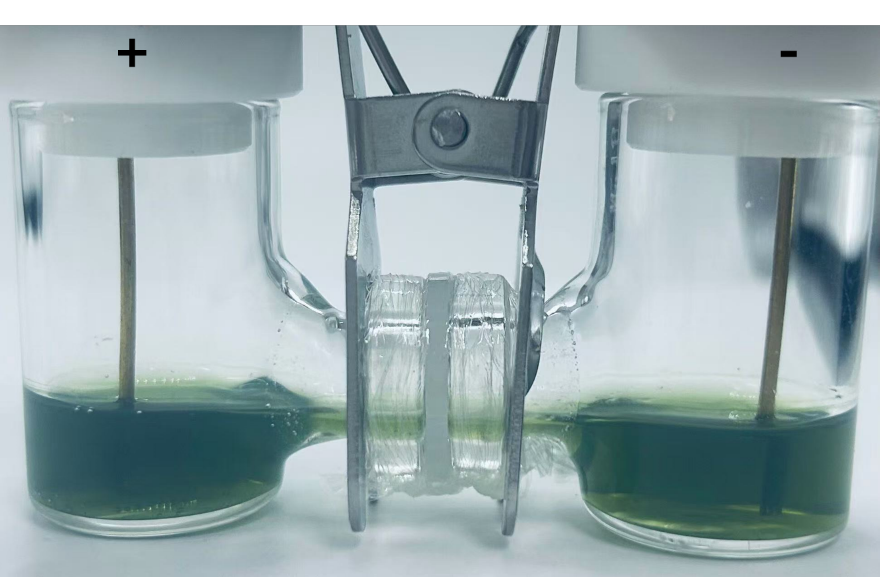


**Figure S1** Device for investigating Chl aggregation at the positive electrode.


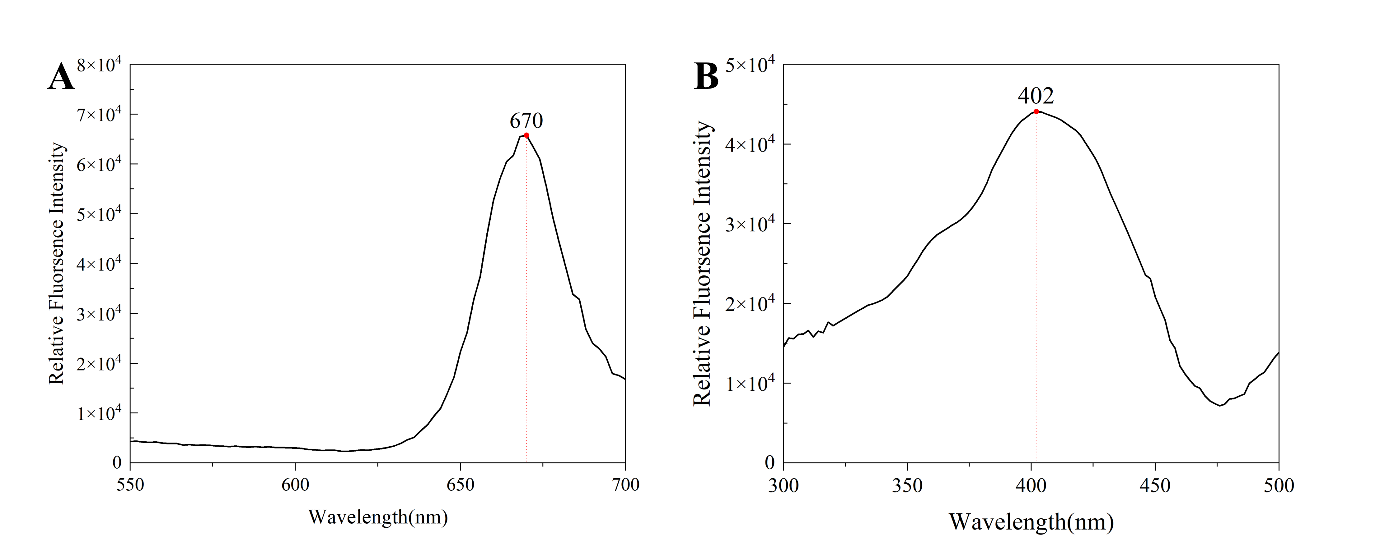


**Figure S2** Determination of measurement parameters for fluorescence emission spectra of SCC: fluorescence emission spectrum at an excitation wavelength of 402 nm (A) fluorescence excitation spectrum at an emission wavelength of 670 nm (B).

1. ^†^ These authors contributed equally to this work.

   * Corresponding author. E-mail address: lifangwei@ouc.edu.cn (Fangwei Li). [↑](#footnote-ref-1)
2. * Corresponding author. E-mail address: mingyz@ouc.edu.cn (Mingyong Zeng). [↑](#footnote-ref-2)
